# Influence of light on the infection of *Aureococcus anophagefferens* CCMP 1984 by a “giant virus”

**DOI:** 10.1371/journal.pone.0226758

**Published:** 2020-01-03

**Authors:** Eric R. Gann, P. Jackson Gainer, Todd B. Reynolds, Steven W. Wilhelm

**Affiliations:** 1 Department of Microbiology, University of Tennessee, Knoxville, Tennessee, United States of America; 2 Department of Biology, Tennessee Wesleyan University, Athens, Tennessee, United States of America; Columbia University, UNITED STATES

## Abstract

The pelagophyte *Aureococcus anophagefferens* has caused recurrent brown tide blooms along the northeast coast of the United States since the mid-1980’s, and more recently spread to other regions of the globe. These blooms, due to the high cell densities, are associated with severe light attenuation that destroys the sea grass beds which provide the basis for many fisheries. Data collected by transmission electron microscopy, PCR, and metatranscriptomic studies of the blooms, support the hypothesis that large dsDNA viruses play a role in bloom dynamics. While a large (~140 nm) icosahedral virus, with a 371 kbp genome, was first isolated more than a decade ago, the constraints imposed by environmental parameters on bloom infection dynamics by Aureococcus anophagefferens Virus, (AaV) remain unknown. To investigate the role light plays in infection by this virus, we acclimated *A*. *anophagefferens* to light intensities of 30 (low), 60 (medium) or 90 μmol photons m^-2^ s^-1^ (high) and infected cultures at these irradiance levels. Moreover, we completed light shift experiments where acclimated cultures were exposed to even lower light intensities (0, 5, and 15 μmol photons m^-2^ s^-1^) consistent with irradiance found during the peak of the bloom when cell concentrations are highest. The abundance of viruses produced per lytic event (burst size) was lower in the low irradiance acclimated cultures compared to the medium and high acclimated cultures. Transferring infected cultures to more-limiting light availabilities further decreased burst size and increased the length of time it took for cultures to lyse, regardless of acclimation irradiance level. A hypothetical mechanism for the reduced efficiency of the infection cycle in low light due to ribosome biogenesis was predicted from pre-existing transcriptomes. Overall, these studies provide a framework for understanding light effects on infection dynamics over the course of the summer months when *A*. *anophagefferens* blooms occur.

## Introduction

The pelagophyte *Aureococcus anophagefferens* has caused recurrent brown tide blooms off the eastern coast of the United States since 1985 [1], where blooms can achieve over 10^6^ cells mL^-1^ during summer months [2]. These blooms typically occur when dissolved inorganic nutrient levels decline, and dissolved organic nutrient levels increase [3]. Although not known to produce compounds toxic to humans, *A*. *anophagefferens* blooms cause severe light attenuation that can kill the sea grass beds which provide valuable nurseries and refuge for fish. It is also believed that *A*. *anophagefferens* may produce compounds toxic to bivalves [4]. While blooms were once thought to be constrained to the northeastern seaboard of the USA, they are now becoming a global problem with *A*. *anophagefferens* spreading down the US eastern coast [5], as well as to other countries [6, 7], possibly *via* transport in ballast water of ships [8].

During the initial characterization of blooms, natural populations of *A*. *anophagefferens* were shown, by transmission electron microscopy (TEM), to be infected with large (~140 nm) virus particles [1]. Later, TEM studies revealed low percentages of infected cells within natural populations during the onset and peak of the bloom, but during bloom collapse over one-third of the natural *A*. *anophagefferens* population were infected [9]. A virus consistent in both size (~140 nm) and morphology (icosahedral) to both the initial and subsequent studies of the bloom was isolated [10–12]. The Aureococcus anophagefferens Virus, AaV, belongs to the algal branch of the *Mimiviridae* family within the Nucleocytoplasmic large dsDNA viruses group [12]. AaV is considered to be a giant virus due to its large particle size, as well as the large genome (371 kbps) it contains [12]. Algae-infecting *Mimiviridae* similar to AaV have been detected by PCR over the course of brown tide blooms [13], and transcripts of viral origin are detected within bloom meta-transcriptomes [14], suggesting this virus to be relevant to understanding the natural populations infecting the blooms. As in several other marine algal blooms (*e*.*g*., [15]), it is hypothesized that viruses infecting *A*. *anophagefferens* play an important role in bloom dynamics and collapse.

To constrain the bloom, viruses infecting *A*. *anophagefferens* must produce enough progeny to remain infectious to lyse the entire population of cells and maintain infectious populations at high enough abundances to encounter susceptible host strains. Changes in productivity (*i*.*e*. burst size or length of infection cycle) of viral infections and decay rates of free virus particles modulate the dynamics of the host viral system. Many studies in the laboratory and the environment have shown certain environmental factors cause increased viral decay rates although these are virus specific [16, 17], but how the changing environment influences the infection cycle within a cell is less understood. Abiotic factors such as irradiance level [18, 19], temperature [20], and nutrients [21] have been shown to negatively influence the infection cycle dynamics in various algal virus systems. For *A*. *anophagefferens*, it is known that decreased temperatures and irradiance increase the time it takes for cultures to lyse, although there was no report of effects on viral abundances [22]. Moreover, increasing the multiplicity of infection (MOI) causes a reduction in the burst size in cultures [23].

Lab experiments of *A*. *anophagefferens* have shown irradiance levels influence growth [24], transcription of many core metabolic pathways [25], and alter uptake of nutrients [26]. The current study aimed to understand the effects of irradiance levels on the infection cycle that were caused by changes to the *A*. *anophagefferens* cell. This is relevant to the *A*. *anophagefferens* system as there is severe light attenuation over the course of the bloom [27], and *A*. *anophagefferens* is well adapted for low light [3, 24]. These culture-based studies provide a framework for the changing host virus dynamics that occur on a population level over the summer months (as increased cell concentration increases light attenuation). Specifically, we examined how the length of the infection cycle and burst size varied in cultures acclimated to decreased irradiance levels. Moreover, as the light field shifts during the growing season, we completed mock-shift experiments consistent with light attenuation in the bloom to determine how decreases in light availability during infection could shape infection outcome. Finally, we determined differences in infection dynamics in populations transitioning from one physiological state to another due to shifting light compared to pre-acclimated populations. These results illustrate the importance of considering the varying irradiance levels at an individual or population level and the impact on infection cycle dynamics.

## Methods

### Culture conditions

Non-axenic *Aureococcus anophagefferens* CCMP1984 was grown at 19° C, on a 14:10 light dark cycle in modified ASP_12_A growth medium [28]. Cultures were acclimated to three different irradiance levels (high: 90 μmol photons m^-2^ s^-1^, medium: 60 μmol photons m^-2^ s^-1^, and low: 30 μmol photons m^-2^ s^-1^) for at least three successive transfers at each irradiance level prior to any experiments. *A*. *anophagefferens* concentrations were determined using a GUAVA-HT6 flow cytometer (MilliporeSigma, Burlington, MA) with abundances for cells gated on red chlorophyll fluorescence and forward scatter. Mean forward scatter (FSU-H) for the *A*. *anophagefferens* gated population was also recorded. Cell concentrations were either measured directly after sampling, or first fixed in 0.5% glutaraldehyde (for the one-step Experiment) and stored at 4° C before being measured within 8 h [11]. Cultures (20 mL) were infected with fresh AaV lysate that was concentrated using a Lab-scale Tangential Flow Filtration System (Fisher Scientific, Waltham, USA) equipped with a Durapore^™^ 30kDa Pellicon XL Filter (MilliporeSigma, Burlington, MA). Concentrates were passed through a 0.45-μm pore-size syringe filter (Millex-HV 0.45μm nominal pore-size PVDF, Millipore Sigma, Burlington, USA) before introduction to cultures at a multiplicity of infection (MOI) of ~100 total particles per cell, which is ~1 infectious particle per cell (see below).

### Plaque assay

Assessment of infectious virus particles was determined using a previously described plaque assay [29, 30]. The bottom agar was ASP_12_A with 1% low melting agarose (Fischer Scientific, Waltham, USA), while the top agar was ASP_12_A with 0.4% low melting agarose. Both were sterilized by Tyndallization. Each plate required ~50 mL of concentrated one-week old *A*. *anophagefferens* culture. *A*. *anophagefferens* cultures were pelleted by centrifugation (2000xg, 5 minutes), and resuspended in ASP_12_A to concentrate the cells fifty-fold. 100 μL of diluted AaV were mixed with 900 μL concentrated *A*. *anophagefferens* and immediately 3 mL of top agar at 30° C was added and poured onto 18 mL of solidified bottom agar. Plates were incubated at 19° C on a 14:10 light dark cycle at an irradiance of 90 μmol photons m^-2^ s^-1^ for one to two weeks in a sealed plastic bag with a damp paper towel to prevent the plates from drying out. Plaques were enumerated and the concentration of plaque forming units determined using the following equation:

$$\frac{PFUs}{mL}=\frac{number\;of\;plaques}{Dilution\times volume\;plated}$$

### Most probable number assay

A most probable number (MPN) assay for *A*. *anophagefferens* was developed in parallel with the plaque assay as an independent determination of the number of infectious virus particles [31]. Aliquots from one-week old *A*. *anophagefferens* cultures (150 μL) were dispensed into round bottom 96-well plates (Corning, Corning, USA). AaV was serially diluted (at either 1:4 or 1:5 dilutions) twelve times. The final row for each plate was serially diluted with sterile growth medium as a non-infected control. Plates were incubated at 19° C on a 14:10 light dark cycle at an irradiance of 90 μmol photons m^-2^ s^-1^ for one to two weeks in a sealed plastic bag with a damp paper towel to prevent drying out. *A*. *anophagefferens* cells in wells were enumerated using in a Cytation 5 plate reader (BioTek, Winooski, USA) set to excitation λ = 614 nm, emission λ = 670 nm as a proxy for biomass within each well. Wells that contained cultures that lysed completely had comparable readings to growth media alone under these settings. The most probable number of infectious particles was calculated by comparing lysed *vs*. unlysed wells at each dilution using previously developed software [32].

### Enumeration of free AaV particles by quantitative PCR

A quantitative PCR (qPCR) assay was developed to enumerate gene copy number for the viral major capsid protein (MCP). The forward primer, MCP_F, (5’-TGGATGCACATCTGGAA) was positioned where degenerate primers were designed to amplify all known algal *Mimiviridae* members [13]. The forward and reverse primer, MCP_R3 (5’- CAATAAGGGGAAGGGCAAG), amplifies a 196 base pair product that is specific to AaV, as confirmed by sequencing. Reaction mixtures for PCR contained: 2 μL of 0.45m-filtered (Millex PVDF syringe filter, MilliporeSigma, Burlington, USA) AaV lysate, 0.5 μL of 100 mM MCP_F, 0.5 μL of 100 mM MCP_R3, 12.5 μL ABsolute qPCR SYBR Green Mix (Thermo Fisher Scientific, Waltham, USA), and 9.5 μL sterile Milli-Q water. The reaction was performed on a DNA Engine Opticon 2 (Bio-Rad, Hercules, USA) using the following conditions: (i) 95° C for 10 min, (ii) 95° C for 30 sec, (iii) 55° C for 30 sec, (iv) 72° C for 30 sec, (v) repeat II–IV 30 times, (vi) 72° C for 10 min, hold at 4° C. Threshold cycle numbers were determined using Opticon Monitor 3 (Bio-Rad, Hercules, USA).

To convert threshold cycle number to absolute gene copy number the 196 base pair product from the above primers was ligated into the pCR 4-TOPO vector (Thermo Fisher Scientific, Waltham, USA) using the 3’ A overhangs generated by the PCR reaction, and transformed *via* heat shock into One Shot TOP10 chemically competent *E*. *coli* (Thermo Fisher Scientific, Waltham, USA). Plasmids were purified using the QIAprep Spin Mimiprep Kit (Qiagen, Hilden, Germany), and the concentration of DNA was quantified using a Nanodrop ND-1000 spectrophotometer (Thermo Fisher Scientific, Waltham, USA). For each qPCR plate, reactions containing 10-fold serial dilutions of the purified circular plasmid were also run as standards. The logarithmic trendline of the diluted standards was then used to convert threshold cycle number to copies of MCP.

Burst sizes were calculated from infection experiments by first determining the difference between the stable maximum (time point B) and minimum (time point A) virus densities using. In our experiments in section ‘Effects of pre-infection light intensities on AaV virus production’, this corresponded to days 1 and 3. For our experiments in section ‘Effect of reduced light availability post infection’ this corresponded to days 1 and 2 for all treatments except the low light acclimated culture maintained at 30 μmol photons m^-2^ s^-1^, which was days 1 and 3. We then normalized these data to viruses per cell values (i.e., burst size) based on the abundance of cells lysed. In total the following equation was used:

$$burst\;size=\frac{virus\;concentration\;B-virus\;concentration\;A}{cell\;concentration\;A-cell\;concentration\;B}$$

### Enumeration of total virus particles

Virus particle densities were enumerated by epifluorescence microscopy as described previously [33]. Briefly, virus particles were separated from *A*. *anophagefferens* by filtration (Millex-HV 0.45μm PVDF syringe filter; MilliporeSigma, Burlington, USA). A working solution of SYBR Green DNA stain (Lonza, Basel, Switzerland) and an anti-fade solution were made fresh and stored in the dark before microscopy. To create the working stock of SYBR Green, it was diluted 400-fold in Milli-Q H_2_O, and then filter sterilized (Nalgene 0.2μm SFCA Syringe Filter, Thermo Fisher Scientific, Waltham, USA). Anti-fade solution was made by diluting a filter sterilized (Nalgene 0.2μm SFCA Syringe Filter, Thermo Fisher Scientific, Waltham, USA) 1% w/v p-phenylenediamine in an autoclaved 50%—PBS/ 50%—Glycerol solution [33]. Viruses were collected on a 25-mm diameter, 0.02-μm Anodisc filter (Whatman, Maidstone, UK). Anodiscs were placed on 18 μL of diluted SYBR green and incubated for ten minutes in the dark. The filters were placed between 36 μL of the anti-fade solution on a glass slide and glass cover slip. Random views were counted on a Leica DM5500 microscope at 1000x magnification with a L5 filter cube (Leica, Wetzler, Germany) per slide to determine the number of viruses per grid. The concentration of virus particles was determined by averaging the random views together and accounting for the size of the grid using the following formula:

$$\frac{Viruses}{mL}=P_{f}*\frac{A_{a}}{A_{g}*V_{f}}*D$$

Where P_f_ is the average viruses counted per field, A_a_ is the total filterable area of the Anodisc filter, A_*g*_ is the area of the eyepiece grid, V_f_ is the volume filtered, and D is the dilution factor.

### Bioinformatic predictions for effects of light on infection

We explored publicly available transcriptomics data to look for a mechanistic link between virus-effects and the results described below. We accessed a previous *de novo* transcriptome which addressed differences between 100 μmol photons m^-2^ s^-1^ and 30 μmol photons m^-2^ s^-1^ acclimated cultures of *Aureococcus anophagefferens* CCMP1850 [25]. Genes that were significantly (Analysis of Sequence Counts [34], p < 0.05) differentially expressed in the low light *vs*. high light conditions and had the best BLAST hit to the *Aureococcus anophagefferens* CCMP1984 genome were compared to the significantly (edgeR [35], FDR p < 0.05) differentially expressed genes from a transcriptome of *Aureococcus anophagefferens* CCMP1984 over an AaV infection cycle [11] to determine overlap. Predicted function and cellular processes associated with each gene were assigned using KEGG pathways [36].

### Statistics

Statistical analyses were performed in Prism 7.03 (GraphPad, San Diego, USA) or R software v. 3.4.0 [37]. Differences between doubling time and burst sizes in cultures with different acclimation irradiance levels were analyzed using a one-way ANOVA followed by Tukey’s HSD post-hoc testing. To determine differences in enumeration methods (qPCR, SYBR green staining, MPN) we utilized a single time point in an experiment (see Effects of pre-infection light intensities on AaV virus production), to enumerate viruses by all three methods. This point was chosen as the epifluorescence counts were not statistically different from one another (S1 Table). The effects of treatment and enumeration method were analyzed by two-way ANOVA. Post-hoc multiple comparisons were adjusted with Tukey’s HSD. The adjusted p-values are reported in S1 Table. Differences between treatments in the post-infection light shift experiment were analyzed using a one-way ANOVA followed by Tukey’s HSD post-hoc testing. Adjusted p-values are reported in S2 Table.

## Results

### Infectivity vs particle abundance of Aureococcus anophagefferens Virus particles

**Fig 1:**
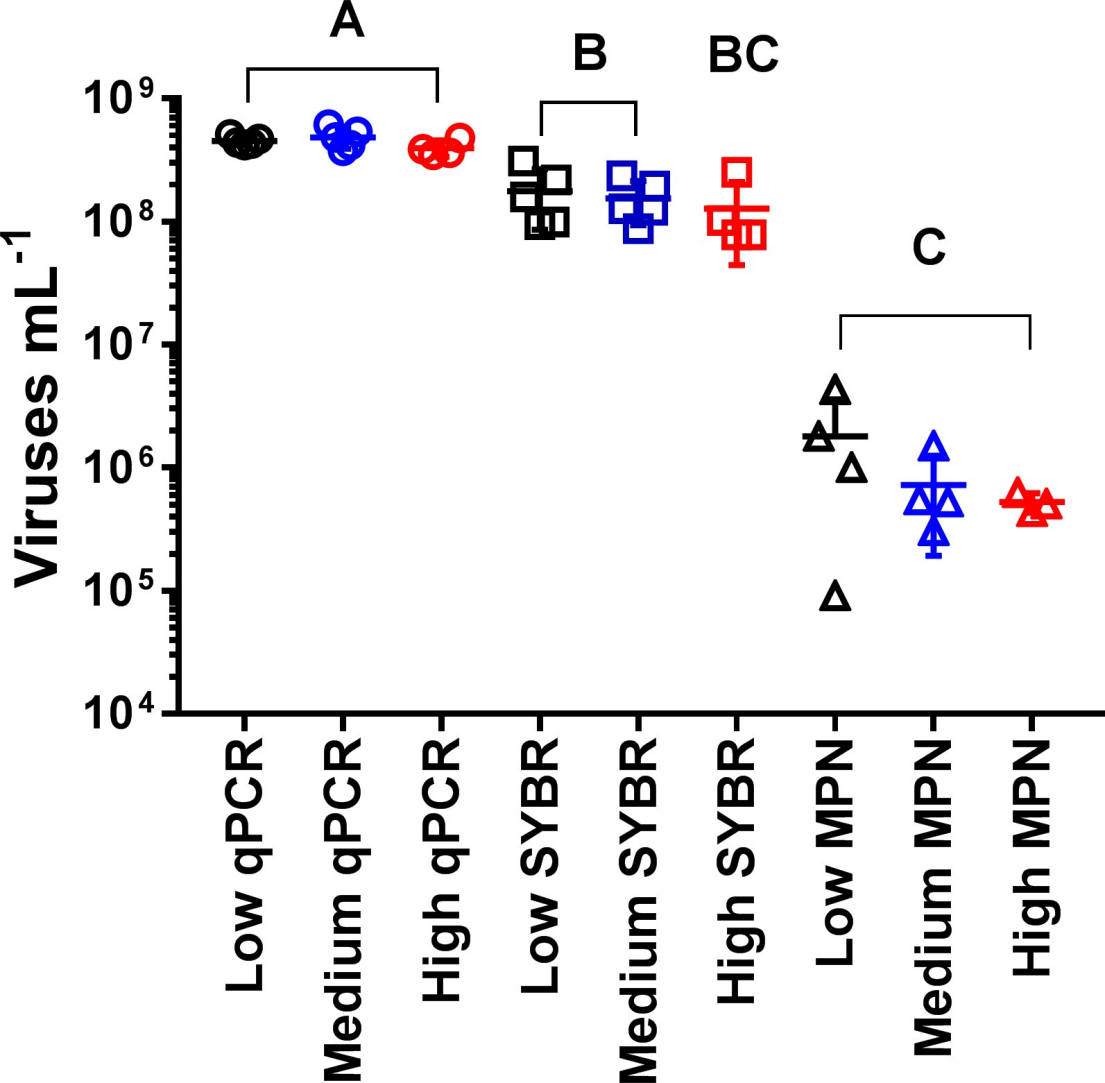
Comparison of three different methods to enumerate AaV. Epifluorescence microscopy with SYBR green, qPCR, and most probable number counts were used to enumerate virus concentration 13 days post infection of cultures acclimated to three different irradiance levels (high: 90 μmol photons m^-2^ s^-1^, medium: 60 μmol photons m^-2^ s^-1^, and low: μmol photons m^-2^ s^-1^). Letters represent statistically indistinguishable concentrations (two-way ANOVA; see Methods and S1 Table).

As part of this study, we first needed to develop protocols to enumerate both the total concentration of AaV particles, as well as the infectious fraction of the particles or those that are able to produce an active infection one adsorbed to the cell. In the developed qPCR assay, there was a linear relationship between the threshold cycle and the abundance of free viruses determined by epifluorescence microscopy (C(t) = -1.492 * ln(viruses mL^-1^) + 36.372, R^2^ = 0.998) to a limit of detection between 1.5–15 viruses μL^-1^ (S1 Fig). In our hands, ~1% of AaV particles enumerated by epifluorescence microscopy were infectious as determined by either the most probable number (MPN) (0.77% infectious, SD = 0.12%) or plaque assay (1.76% infectious, SD = 0.28%) (S2 Fig). We used a single time point in an experiment (see Effects of pre-infection light intensities on AaV virus production) to validate our qPCR assay by also enumerating virus particles by epifluorescence microscopy, as well as by MPN. There was no statistically significant interaction between the effects of the treatment (acclimation irradiance level) and viral enumeration method on the number of viruses counted (Two-way ANOVA; F(4,30) = 0.678, p-value = 0.613). The treatment did not strongly influence the viral counts (Two-way ANOVA: F(4,30) = 0.678, p-value = 0.275), while the viral enumeration method did (Two-way ANOVA: F(4,30) = 0.678, p-value = <0.0001). qPCR counts were higher (average 3.42x, SD = 0.24) than the microscopy counts for each the three different irradiance acclimated cultures (Fig 1).

### Effects of pre-infection light intensities on AaV virus production

**Fig 2:**
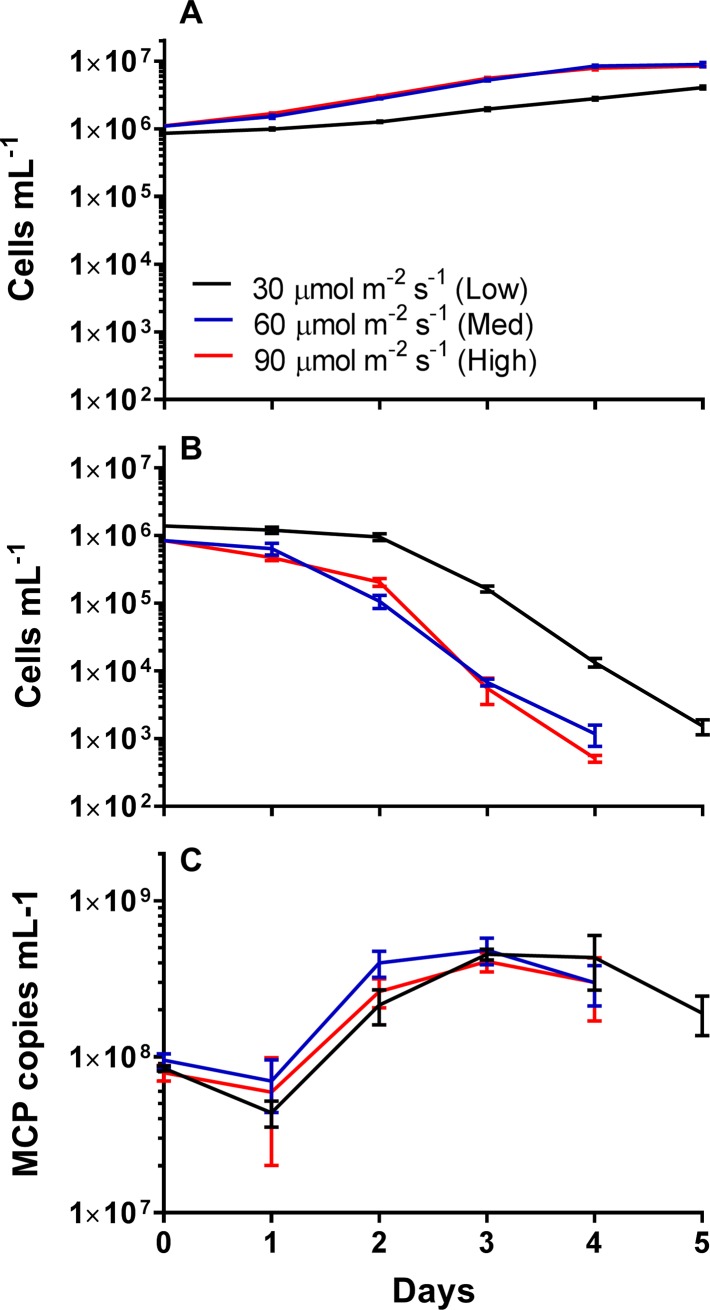
*Aureococcus anophagefferens* virus infection cycle dynamics with acclimation to varying irradiance levels. *A*. *anophagefferens* host concentrations either uninfected (A) or infected (B), and C) major capsid protein (MCP) copies mL^-1^ over the course of the 5-day experiment. Cultures were infected on day 0. Red lines are high irradiance acclimated cultures (90 μmol photons m^-2^ s^-1^), blue lines are medium irradiance acclimated cultures (60 μmol photons m^-2^ s^-1^), and black lines are low irradiance acclimated cultures (30 μmol photons m^-2^ s^-1^). All symbols are for n = 5 five biological replicates ± SD.

*A*. *anophagefferens* cultures were acclimated to three different irradiance levels (high: 90 μmol photons m^-2^ s^-1^, medium: 60 μmol photons m^-2^ s^-1^, and low 30 μmol photons m^-2^ s^-1^). There was no difference in doubling time between medium (1.44 d) and high (1.39 d) irradiance cultures (one-way ANVOA; F = 54.55; p = 0.180), while there was a significant difference in doubling time for low irradiance cultures (1.76 d) compared to the other acclimation irradiance levels (one-way ANOVA; F = 54.55; p < 0.001) (Table 1 and S3 Fig). Compared to uninfected cultures which continued to increase in cell numbers (Fig 2A), cultures infected during late logarithmic growth at these irradiance levels showed no difference in time-to-lysis (Fig 2B and 2C), or burst size (Table 1) for the high light and medium light acclimated cultures (one-way ANOVA; F = 5.3868, p = 0.714). However, there was an increase in the time to complete lysis of the culture (Fig 2B), and a reduction in burst size (Table 1), for the low irradiance condition compared to both medium light (one-way ANOVA; F = 5.3868, p = 0.085) and high light (one-way ANOVA; F = 5.3868, p = 0.021).

**Table 1:** Summary of acclimation conditions including host doubling time (S3 Fig), mean forward scatter (FSU-H) determined by flow cytometry, burst size (MCP copies produced/*A*. *anophagefferens* cell lysed), and percent of particles determined to be infectious (from Fig 1). Standard deviation of each value is recorded within the parenthesis.

Acclimating Irradiance	Low	Medium	High
Uninfected Doubling Time (days)	1.763 (0.06)	1.437 (0.008)	1.387 (0.016)
Uninfected Mean FSU-H (Relative Units)	13.23 (0.58)	18.48 (0.99)	18.40 (1.25)
Burst size (Viruses produced / cells lysed)	335 (149)	670 (201)	761 (181)
Percentage of Particles that are infectious	0.982 (0.545)	0.701 (0.481)	0.480 (0.244)

To determine if the different irradiance treatments influenced the percentage of particles produced during lysis that were infectious, MPN assays were performed three days post infection at a time point when the abundance of virus particles per treatment was not significantly different (Fig 1). For all three light acclimation levels, the percentage of the total particles determined by epifluorescence microscopy that were infectious as determined by MPN were not significantly different from one another (Fig 1, Table 1, and S1 Table). As determined previously (S2 Fig), a low percentage of the total virus population was infectious, with between 0.48 and 0.98% of particles being infectious at that timepoint (Table 1).

### Effect of reduced light availability post infection

**Fig 3:**
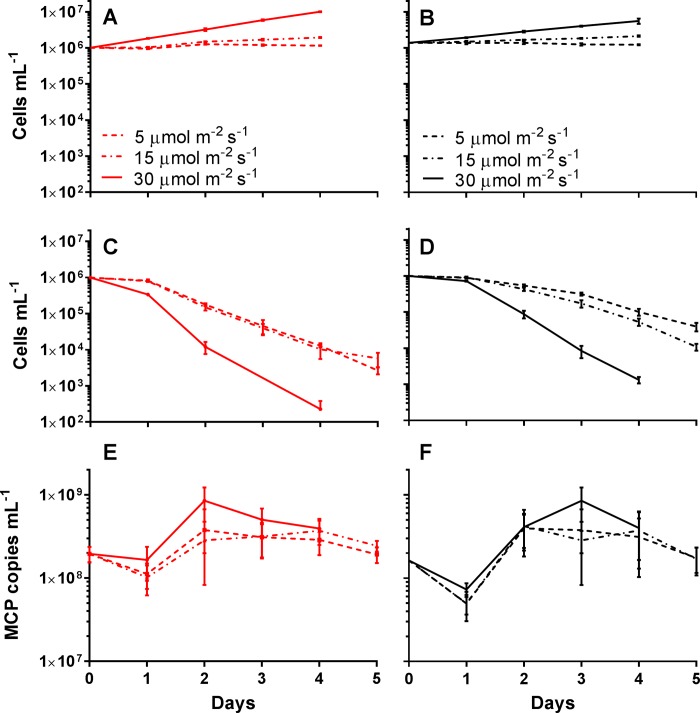
*Aureococcus anophagefferens* virus infection cycle dynamics with acclimation to varying light levels and in lower light. *A*. *anophagefferens* host concentrations for uninfected cultures acclimated to A) 90 μmol photons m^-2^ s^-1^ and B) 30 μmol photons m^-2^ s^-1^, and infected cultures acclimated to C) 90 μmol photons m^-2^ s^-1^ and D) 30 μmol photons m^-2^ s^-1^ over the 5-day experiment. MCP copies mL^-1^ for cultures acclimated to E) 90 μmol photons m^-2^ s^-1^ and F) 30 μmol photons m^-2^ s^-1^ over the 5-day experiment. Cultures were infected on day 0. Solid lines indicate acclimated cultures maintained at acclimated irradiance levels after infection, dotted and dashed lines indicate acclimated cultures transferred into 15 μmol photons m^-2^ s^-1^ light after infection, and dashed lines indicate acclimated cultures transferred into 5 μmol photons m^-2^ s^-1^ light after infection. All symbols are for n = 5 five biological replicates ± SD.

As there were differences in infection cycle dynamics between the high and low irradiance acclimated cultures, the effect of greater light limitation post-infection (light levels of 5 and 15 μmol photons m^-2^ s^-1^) was explored. Light levels were chosen as they are relevant to populations late in the blooms when high cell densities occur [27], as well as for a reduction in light that occurs while individual cells are sinking. These light levels were also determined to limit growth of uninfected cultures, as cell densities had not increased 4 d after being shifted to the decreased light (Fig 3A and 3B). When high or low irradiance acclimated cultures were infected and then transferred into either 15 or 5 μmol photons m^-2^ s^-1^, cultures still maintained a productive infection. Temporally it took two days longer for ~99% of high irradiance acclimated cells to lyse in both limiting light conditions compared to infected cultures maintained at acclimated irradiance levels (Fig 3C). Virus production was observed for the first 2 days after the shift, but then virus abundance remained static (2.4 x 10^8^ MCP copies mL^-1^) (Fig 3E). Similar trends were seen with low irradiance acclimated cultures (Fig 3D and 3F). The lytic cycle was extended for cultures transferred into limiting light compared to the infected cultures maintained at acclimated irradiance levels (Fig 3D), as well as no further increase in viral abundance 2 days post infection (Fig 3F).

**Fig 4:**
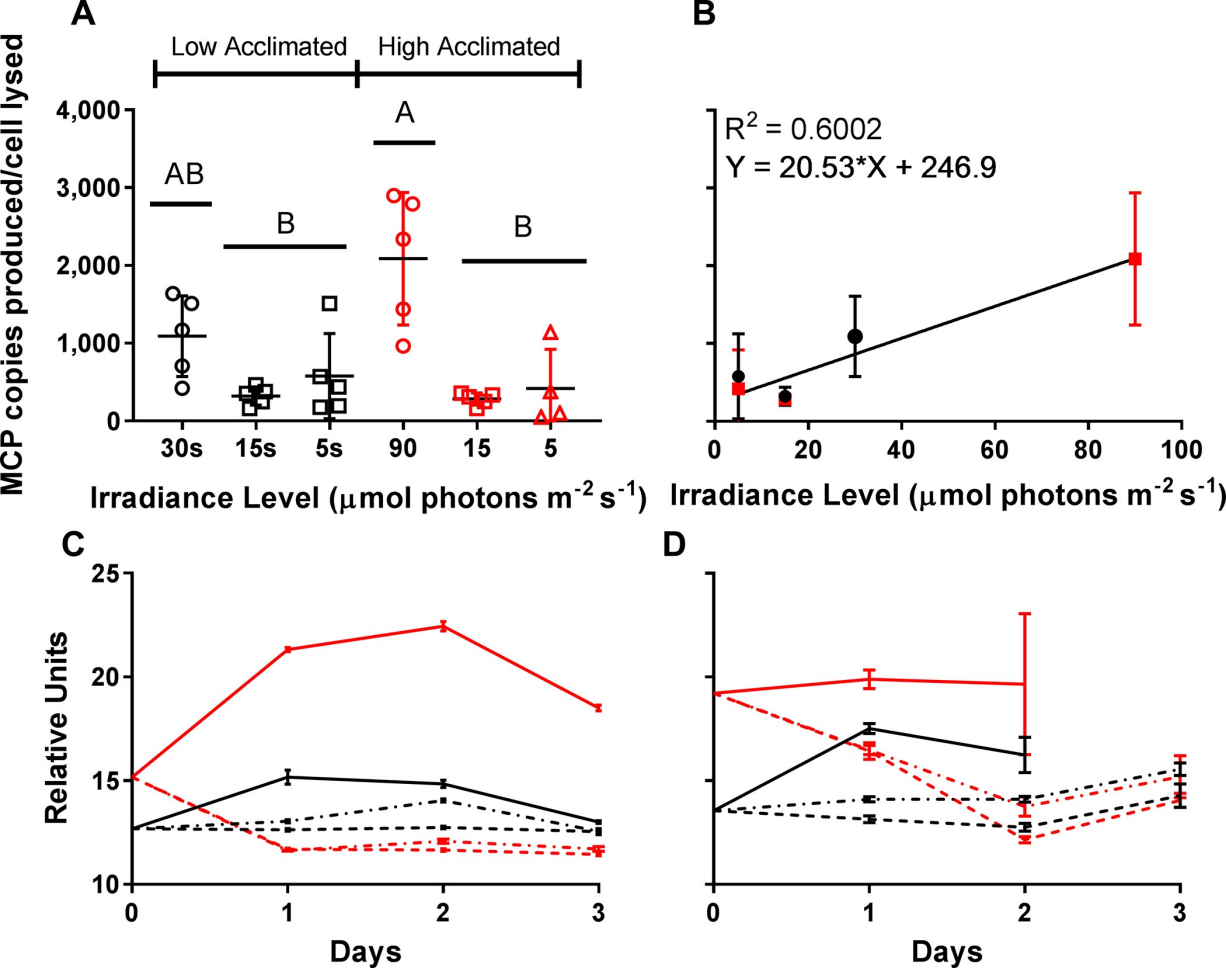
Effects of reducing light levels to 15 and 5 μmol photons m^-2^ s^-1^ after infection on Burst Size (Fig 3). Red Lines and symbols are high irradiance acclimated (90 μmol photons m^-2^ s^-1^), while black lines are low irradiance acclimated (30 μmol photons m^-2^ s^-1^). A) MCP copies produced per cell lysed for each treatment. Letters represent statistically indistinguishable burst sizes (one-way ANOVA; see Methods and S2 Table). B) Burst Size v. Irradiance Level during the infection. C) Mean forward scatter of uninfected *A*. *anophagefferens* cultures over the first three days of the 5-day experiment (Fig 2C, Fig 2D) as determined by flow cytometry. D) Mean forward scatter of infected *A*. *anophagefferens* cultures over the course of the 5-day experiment (Fig 2C, Fig 2D) as determined by flow cytometry. Plotted are only populations >10,000 cells/mL. Solid lines indicate acclimated cultures maintained at acclimated irradiance levels after infection, dotted and dashed lines indicate acclimated cultures transferred into 15 μmol photons m^-2^ s^-1^ light after infection, and dashed lines indicate acclimated cultures transferred into 5 μmol photons m^-2^ s^-1^ light after infection. Error is plotted as standard deviation.

The number of viruses produced per lytic event was influenced by the irradiance level at which the infection occurred. When cultures started at high (90 μmol photons m^-2^ s^-1^) or low (30 μmol photons m^-2^ s^-1^) irradiance levels were transferred to 15 or 5 μmol photons m^-2^ s^-1^ conditions the burst sizes were not significantly different from one another, despite initially different irradiance levels (Fig 4A, S2 Table). Cultures shifted to limiting light levels had a pronounced reduction in burst size, averaging 19.15% (SD = 16.67%) of the high irradiance cultures infected and maintained at acclimating irradiance levels (Fig 4A, S2 Table). A linear relationship (burst size = 20.53* irradiance level + 246.9, R^2^ = 0.600) between burst size and irradiance level was seen (Fig 4B). We note that while between different experiments trends we observed were conserved, similar treatments sometimes gave variable results. One example of this is that there is a significant difference between both 30 μmol photons m^-2^ s^-1^ (unpaired t-test between 30 μmol photons m^-2^ s^-1^ burst sizes from Table 1 and Fig 4A: p = 0.019) and 90 μmol photons m^-2^ s^-1^ (unpaired t-test between 90 μmol photons m^-2^ s^-1^ burst sizes from Table 1 and Fig 4A: p = 0.0095). Yet, when burst size is normalized to the 90 μmol photons m^-2^ s^-1^ cultures, the linear relationship between burst size and irradiance level is still maintained (S4 Fig).

It appeared there were two phases of viral production in cultures transferred to lower light post infection. The first phase was characterized by cells lysing concurrent with an increase in free virus concentration (Day 0–2) (Fig 3D and 3F), while in the second phase the decline in *A*. *anophagefferens* cells did not produce more viruses. Over the course of the first two days the mean forward scatter from the flow cytometric estimates of the population decreased before remaining constant for the duration of the experiment for both the uninfected (Fig 4C), and infected populations (S3D Fig).

Finally, to determine if the infection cycle required light to complete, we infected high, medium, and low irradiance acclimated cultures and transferred them into the dark. There was no increase in host cell concentration in uninfected cultures (S5A Fig), unlike cultures kept at the acclimation irradiance (Fig 2A), nor was there a large decrease in infected cultures (S5B Fig). This lack of cell death was accompanied by no increase in virus concentration in any of the cultures regardless of light acclimation condition (S5C Fig). Virus abundance decreased to 0.16–1.61% of starting concentration by day 10.

### Effects of pre-infection light reduction on the progress of AaV infection

**Fig 5:**
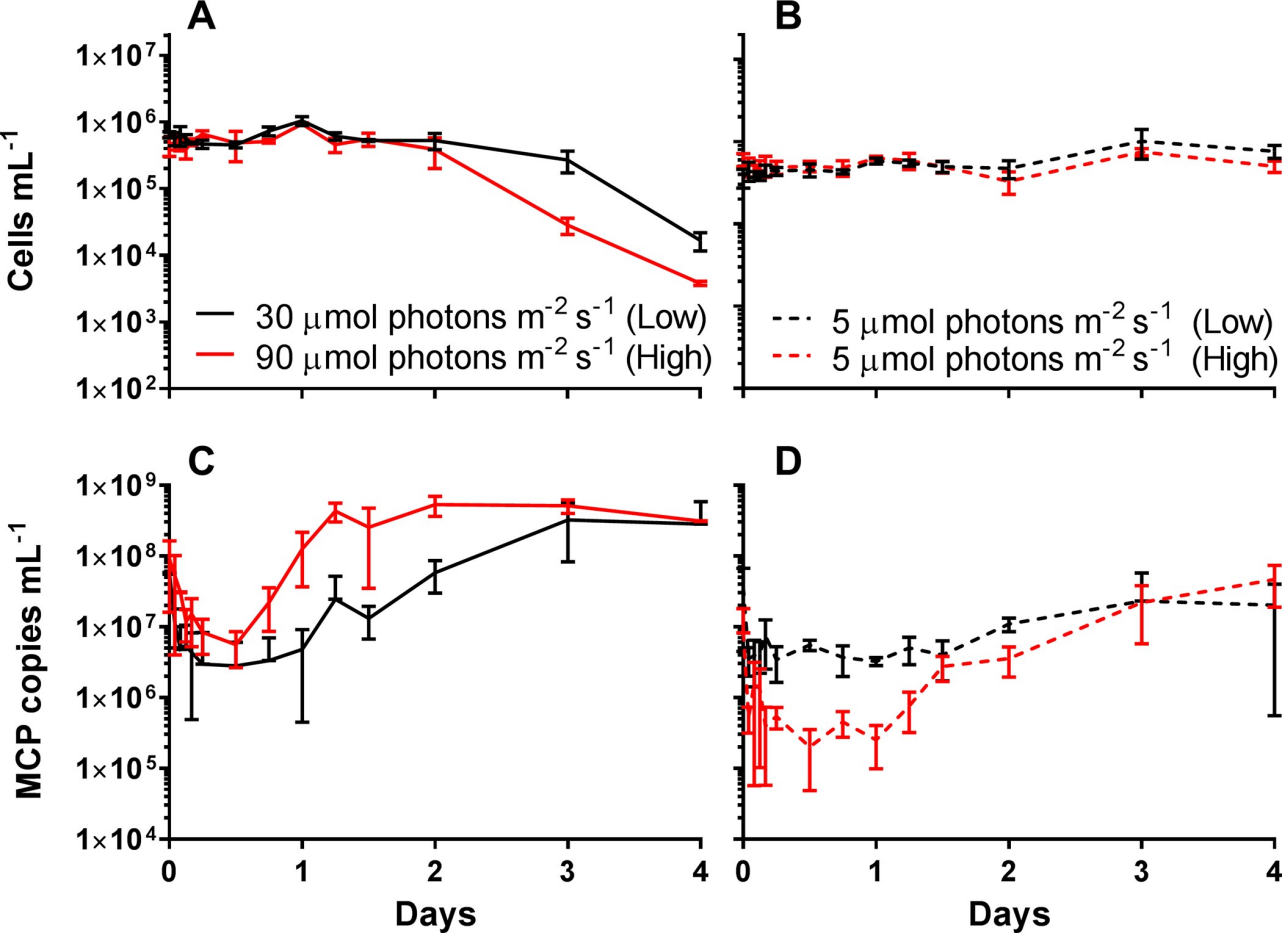
*Aureococcus anophagefferens* virus infection cycle dynamics with pre-acclimation to 5 μmol photons m^-2^ s^-1^ for one day before infection. Red lines are high irradiance acclimated (90 μmol photons m^-2^ s^-1^) and black lines are low irradiance acclimated (30 μmol photons m^-2^ s^-1^). *A*. *anophagefferens* host concentrations for acclimated cultures A) maintained at acclimated irradiance levels after infection or B) infected after one day at 5 μmol photons m^-2^ s^-1^. MCP copies mL^-1^ for acclimated cultures C) maintained at acclimated irradiance levels after infection or D) infected after one day at 5 μmol photons m^-2^ s^-1^. Day 0 on the graphs are when cultures were infected. Points are for n = 3 three biological replicates ± SD.

To determine whether this transition from irradiance levels where the cells could actively grow to where they only persisted (Fig 3A and 3B) caused the different phases of the infection cycle we observed, cultures acclimated to either high or low irradiance levels were pre-acclimated to 5 μmol photons m^-2^ s^-1^ for one day to allow the population to shift to the altered physiological state, as determined by a decrease in forward scatter (S3 Table). Cultures were then infected at this limiting irradiance acclimation. *A*. *anophagefferens* concentrations decreased over the four day experiment in cultures that were kept at their acclimation irradiance (Fig 5A), while there was no decrease in host concentration in cultures pre-acclimated and infected at 5 μmol photons m^-2^ s^-1^. The infection cycle of the limiting irradiance acclimated cultures was determined to be longer (24–30 h) than those cultures maintained at high irradiance (12–18 h) (Fig 5C), as determined by an increase in free virus concentration. The cultures pre-acclimated to the 5 μmol photons m^-2^ s^-1^ of light for one day did not show an increase in viruses within the first two days post infection as in the experiment where cultures were not pre-acclimated (Figs 3E, 3F and 5D). Free virus concentration increased after 30–36 h for high irradiance acclimated cultures that were pre-acclimated to limiting irradiance, while low irradiance acclimated cultures that were pre-acclimated to limiting irradiance produced new viruses between 36–48 h (Fig 5D).

### Informatic examination of the effects of light and virus infection

**Fig 6:**
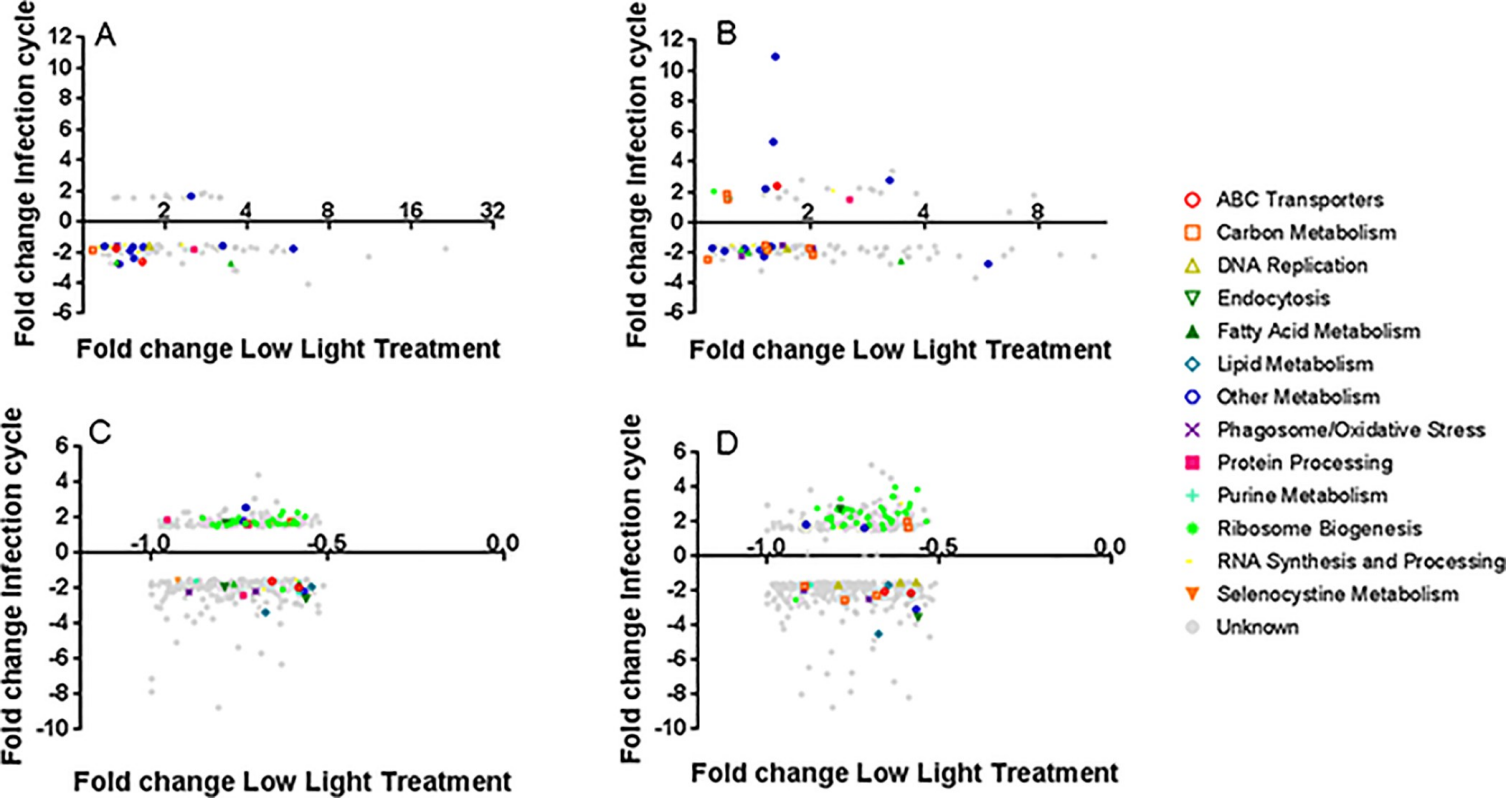
Comparison between *de novo* assembled *Aureococcus anophagefferens* CCMP1850 transcriptome in low light [25] and *Aureococcus anophagefferens* CCMP1984 transcriptome over the course of the infection cycle [11]. A) Significantly overexpressed genes detected in Frischkorn *et al*. Transcriptome compared to genes significantly over expressed during the early stages of the infection cycle (5 min, 30 min, 1 hr, 6 hr). B) Significantly overexpressed genes detected in Frischkorn *et al*. Transcriptome compared to genes significantly over expressed during the late stages of infection cycle (12 hr and 21 hr). C) Significantly underexpressed genes detected in Frischkorn *et al*. Transcriptome compared to genes significantly over expressed during the beginning of infection cycle (5 min, 30 min, 1 hr, 6 hr). D) Significantly underexpressed genes detected in Frischkorn *et al*. Transcriptome compared to genes significantly over expressed during the late stages of infection cycle (12 hr and 21 hr). The value for the fold change over the course of the infection cycle is the average of significantly differentially expressed fold change for either the early stages of infection (A, C) or the late stages of infection (B, D).

To develop hypotheses concerning processes in *A*. *anophagefferens* that are different in various acclimated cultures, publicly available transcriptomic data were screened to compare the potential effects of light and the effects of the infection cycle. Published transcriptomes of *Aureococcus anophagefferens* CCMP1850 comparing cultures at two light levels (30 and 100 μmol photons m^-2^ s^-1^) were examined [25]. Although that study used a different strain, 95% of their reads mapped to *Aureococcus anophagefferens* CCMP1984. To be more stringent, only assembled reads that had top BLAST hits to CCMP1984 were used [25]. Of 1,524 differentially expressed genes detected in low light *vs*. high light cultures, 49.2% of those genes were also differentially expressed in a transcriptome of *Aureococcus anophagefferens* CCMP1984 infected with AaV at 100 μmol photons m^-2^ s^-1^ in at least one time point [11] (S4 Table). To contrast gene expression between low light the infection cycle transcriptome and infection cycle transcriptomes, KEGG annotations and pathways [36] were examined (S5 Table). Pathways included those for metabolism of sugars, nucleic acids, and amino acids. Host nucleic acid scavenging and recycling have been hypothesized to be required for the AT rich AaV to take advantage of the metabolism of the GC rich *A*. *anophagefferens* [23]. Interestingly, the pathway that showed the strongest differences between the infection cycle and cells in 30 μmol photons m^-2^ s^-1^ was ribosome biogenesis. 13/15 ribosomal genes detected in the KEGG pathway were downregulated in the 30 μmol photons m^-2^ s^-1^ culture compared to 100 μmol photons m^-2^ s^-1^ (S5 Table). We then searched for other genes involved in ribosome biogenesis and found 41 genes that were differentially expressed in our subset of both data sets (Table 2), most of which were upregulated at the later time points of the infection cycle, while being down regulated in the 30 μmol photons m^-2^ s^-1^ transcriptome (Fig 6).

**Table 2:** Subset of the *Aureococcus anophagefferens* genes predicted to be significantly downregulated in low light [25] that are related to ribosome biogenesis according to KEGG classifications. Significant fold change values over the course of the infection cycle transcriptome are shown. Significantly overexpressed are in blue while those in red are significantly under expressed.

		Significant fold change in Infection Cycle Transcriptome					
Accession	KEGG Gene Description	5 min	30 min	1 h	6 h	12 h	21 h
AURANDRAFT_10453	K13179 DDX18; ATP-dependent RNA helicase DDX18/HAS1 [EC:3.6.4.13]	-	-	-	1.86	2.81	2.73
AURANDRAFT_1296	K14787 MRD1; multiple RNA-binding domain-containing protein 1	-	-	-	2.13	3.87	3.07
AURANDRAFT_14446	K14841 NSA1; ribosome biogenesis protein NSA1	-	1.71	-	2.44	4.99	2.77
AURANDRAFT_1519	K14806 DDX31; ATP-dependent RNA helicase DDX31/DBP7 [EC:3.6.4.13]	-	-	-	-	1.95	-
AURANDRAFT_1767	K14859 SSF1_2; ribosome biogenesis protein SSF1/2	-	-	-	1.65	2.87	2.16
AURANDRAFT_19030	K14768 UTP7; U3 small nucleolar RNA-associated protein 7	-	-	-	1.65	1.98	1.69
AURANDRAFT_20690	K12619 XRN2; 5'-3' exoribonuclease 2 [EC:3.1.13.-]	-	-2.07	-	-	-	-
AURANDRAFT_22083	K06943 NOG1; nucleolar GTP-binding protein	-	-	-	-	2.35	1.52
AURANDRAFT_2416	K14777 DDX47; ATP-dependent RNA helicase DDX47/RRP3 [EC:3.6.4.13]	-	-	-	1.66	2.97	2.17
AURANDRAFT_24989	K12823 DDX5; ATP-dependent RNA helicase DDX5/DBP2 [EC:3.6.4.13]	-	-	-	1.99	2.91	2.67
AURANDRAFT_25894	K14553 UTP18; U3 small nucleolar RNA-associated protein 18	-	-	-	1.66	1.96	1.61
AURANDRAFT_26008	K14809 DDX55; ATP-dependent RNA helicase DDX55/SPB4 [EC:3.6.4.13]	-	-	-	1.52	2.48	2
AURANDRAFT_26477	K14776 DDX10; ATP-dependent RNA helicase DDX10/DBP4 [EC:3.6.4.13]	-	-	-	-	2.1	-
AURANDRAFT_26879	K14847 RPF2; ribosome production factor 2	-	-	-	2.01	2.84	2.48
AURANDRAFT_26912	K14811 DBP3; ATP-dependent RNA helicase DBP3 [EC:3.6.4.13]	-	-	-	1.65	3.18	1.97
AURANDRAFT_27316	K11884 PNO1; RNA-binding protein PNO1	-	-	-	2.35	5	3.02
AURANDRAFT_283	K14569 BMS1; ribosome biogenesis protein BMS1	-	-	-	-	1.64	1.9
AURANDRAFT_28540	K07178 RIOK1; RIO kinase 1 [EC:2.7.11.1]	-	-	-	-	3.44	1.99
AURANDRAFT_31375	K14780 DHX37; ATP-dependent RNA helicase DHX37/DHR1 [EC:3.6.4.13]	-	-	-	-	2.21	1.83
AURANDRAFT_33432	K19306 BUD23; 18S rRNA (guanine1575-N7)-methyltransferase [EC:2.1.1.309]	-	-	-	1.72	2.92	1.59
AURANDRAFT_33949	K07179 RIOK2; RIO kinase 2 [EC:2.7.11.1]	-	-	-	1.58	4.07	2.57
AURANDRAFT_37654	K14775 UTP30; ribosome biogenesis protein UTP30	-	-	-	1.77	3.06	2.64
AURANDRAFT_38045	K14549 UTP15; U3 small nucleolar RNA-associated protein 15	-	-	-	1.9	2.39	2.27
AURANDRAFT_4268	K14843 PES1; pescadillo	-	-	-	1.64	3.98	2.93
AURANDRAFT_4711	K14831 MAK16; protein MAK16	-	-	-	1.67	3.35	2.31
AURANDRAFT_52052	K14857 SPB1; AdoMet-dependent rRNA methyltransferase SPB1 [EC:2.1.1.-]	-	-	-	1.8	2.55	2.41
AURANDRAFT_59367	K14835 NOP2; 25S rRNA (cytosine2870-C5)-methyltransferase [EC:2.1.1.310]	-	-	-	1.61	2.14	2.25
AURANDRAFT_59370	K14824 ERB1; ribosome biogenesis protein ERB1	-	-	-	1.77	2.45	2.09
AURANDRAFT_60066	K14842 NSA2; ribosome biogenesis protein NSA2	-	-	-	1.76	2.45	2.03
AURANDRAFT_60268	K14191 DIM1; 18S rRNA (adenine1779-N6/adenine1780-N6)-dimethyltransferase	-	-	-	2.3	3.45	2.65
AURANDRAFT_62634	K14774 UTP25; U3 small nucleolar RNA-associated protein 25	-	1.5	-	1.78	2.05	1.64
AURANDRAFT_63995	K14557 UTP6; U3 small nucleolar RNA-associated protein 6	-	1.56	-	-	1.85	-
AURANDRAFT_64464	K11883 NOB1; RNA-binding protein NOB1	-	-	-	-	2.59	-
AURANDRAFT_65930	K14554 UTP21; U3 small nucleolar RNA-associated protein 21	-	1.6	-	1.73	1.71	-
AURANDRAFT_68377	K16912 LAS1; ribosomal biogenesis protein LAS1	-	1.71	-	1.63	-	-
AURANDRAFT_68959	K14572 MDN1; midasin	-	-	-	-	-2.12	-2.92
AURANDRAFT_70174	K07562 NMD3; nonsense-mediated mRNA decay protein 3	-	-	-	1.76	3.2	2.25
AURANDRAFT_71183	K14521 NAT10; N-acetyltransferase 10 [EC:2.3.1.-]	-	-	-	-	-	1.58
AURANDRAFT_71380	K14561 IMP4; U3 small nucleolar ribonucleoprotein protein IMP4	-	-	-	-	2.24	3.05
AURANDRAFT_72347	K14537 NUG2; nuclear GTP-binding protein	-	-	-	1.5	2.47	1.63
AURANDRAFT_72521	K14848 RRB1; ribosome assembly protein RRB1	-	-	-	1.68	2.59	1.81

## Discussion

### The infectivity of Aureococcus anophagefferens Virus particles

Both most probable number and plaque assays were developed to determine the percentage of particles that were infectious, as this percentage varies by both the biological system [38] and assay (*i*.*e*., MPN v. Plaque Assay) employed [31]. From our data, *ca* 1% of the virus particles observed by epifluorescence microscopy were determined to be infectious, a proportion similar to viruses infecting *Heterosigma akashiwo* [39], but much less than other systems [19, 40]. In parallel with epifluorescence derived direct counts, a qPCR assay was developed with hopes of eventually deploying it in environmental systems. There was a significant difference between the two types of direct counts. This was not necessarily surprising, as it has previously been reported that circular plasmid standards can overestimate copies within a sample [41]. Yet while the qPCR approach may have produced a relative overestimate, it did provide a lower detection limit relative to epifluorescence. This sensitivity allowed for detection of free virus particle / genome production at 12–18 h, shorter than the 21–24 h infection cycle previously reported [10], although we cannot rule out that changes in culture conditions in experiments completed over a decade apart may have played a role.

### Effect of light acclimation on the progress of infection

*A*. *anophagefferens* thrives in bloom conditions when irradiance levels are reduced due to high cell densities [27]. The effect of light attenuation on viral production is important from an ecological standpoint as viruses are hypothesized to play a role in bloom collapse [9]. To investigate the influence of irradiance, *A*. *anophagefferens* CCMP1984 was acclimated to high (90 μmol photons m^-2^ s^-1^), medium (60 μmol photons m^-2^ s^-1^), and low (30 μmol photons m^-2^ s^-1^) irradiance levels. The growth rates determined for high and medium acclimated cultures (1.39 and 1.44 d, respectively), were similar (1.61–2.04 d) to those seen in several studies growing *A*. *anophagefferens* at irradiance levels between 90 and 100 μmol photons m^-2^ s^-1^ [25, 26]. In agreement with previous studies [24, 26], there was a reduced growth rate as irradiance level decreased. Doubling times between 1.98 and 2.58 d was reported with cultures growth at similar irradiance levels to the low light acclimation culture in this study [26]. Based on the nitrogen source present the difference between high and low light measured by Pustizzi *et al*. was between 0.10 and 0.53 d [26], which is like the 0.37 d difference in doubling time between the low and high acclimated cultures. Infecting high and medium irradiance acclimated cultures resulted in > 99% of cultures lysing in 3 days, with statistically indistinguishable burst sizes. In the low irradiance acclimated cultures, delayed lysis of > 99% of the culture with a reduced burst size occurred, as seen in other systems [40]. The increased time for lysis of low irradiance acclimated cultures may partially be explained by the increase in the length of the lytic cycle (see next section).

### Effect of growth limiting light on infection

Irradiance during blooms with >5 x 10^5^ cells/mL has been reported between 1.1–12.8 μmol photons m^-2^ s^-1^ [27]. To more robustly determine the effects of these limiting light levels, high and low irradiance acclimated cultures were infected and transitioned to 15 μmol photons m^-2^ s^-1^ and 5 μmol photons m^-2^ s^-1^. Transferring cultures to growth limiting light increased the time of complete lysis of cultures. Burst sizes were reduced during these limiting light shift experiments, and were statistically the same, regardless of acclimation in 30 or 90 μmol photons m^-2^ s^-1^. In the first two days there was a decrease in mean forward scatter of the *A*. *anophagefferens* cells dependent on the final light level of the shift. Algae placed low light have previously been shown to have a lower mean forward scatter, which is hypothesized to be due to a decrease in size [20, 40]. We observed this when decreasing acclimation light levels in our system. It is not clear whether *A*. *anophagefferens* cells are on average smaller when maintained in low *vs*. high light: we note cell size did not change over the course of a 14 d study in the dark [8]. At the least this change in mean forward scatter was indicative of a difference in the physiological state of the entire population, within the first day or two of the experiment compared to the later days. This provided a possible explanation for why free virus abundances do not continue to increase after two days following the shift to lower irradiance levels.

To determine how cells in limiting light respond to viral infection, cultures were transferred to 5 μmol photons m^-2^ s^-1^ for one day before infection. *A*. *anophagefferens* cell concentrations remained static after infection in contrast to cultures shifted to 5 μmol photons m^-2^ s^-1^ post infection. Production of new viruses was delayed in cultures pre-acclimated to 5 μmol photons m^-2^ s^-1^, which differed from those not pre-acclimated. The acclimation irradiance level (30 *vs*. 90 μmol photons m^-2^ s^-1^) did impact the length of the lytic cycle as cells acclimated in high light produced new viruses 6–12 hours earlier, even after pre-acclimation to 5 μmol photons m^-2^ s^-1^.

*A*. *anophagefferens* survives prolonged periods in the dark in culture, but with a previously reported 33% decline in viable cells over time [8]. This is similar to the uninfected cell concentration decline over 5 days (22.34–35.48%). No productive infection occurred when *A*. *anophagefferens* was placed in the dark, regardless of acclimation irradiance. Inhibition in the dark has been hypothesized to be due to energy stores within the cell. Infections of the smaller *Micromonas pusilla* are never successful in the dark, but infections of *Phaeocystis globosa* in the dark are successful if cells were acclimated to 250 μmol photons m^-1^ s^-1^, but not 25 μmol photons m^-1^ s^-1^ [18]. *A*. *anophagefferens* is known to utilize its stores when in darkness. For example, during 14 d in darkness, carbohydrates and proteins per cell are reduced by 60% and 89%, respectively [42]. Although we see no increase in free viral abundance in the dark, AaV concentrations do decline 81.6–89.6% within the first day, suggesting adsorption can occur in the dark which is in contrast to other systems where adsorption is greatly influenced by light [43]. This decrease in AaV may partly be to detritus or heterotrophic bacteria in the culture as well.

### Bioinformatic insight into limitations to the virocell at low light

Preexisting *de novo* transcriptomes in a different *A*. *anophagefferens* strain (CCMP1850) grown in 30 or 100 μmol photons m^-2^ s^-1^ [25] were used to hypothesize pathways that could be inversely transcribed compared to the transcriptome of an infected cell [11]. We found many KEGG pathways [36] that were differentially expressed in both 30 μmol photons m^-2^ s^-1^ and at least at one point in the infection cycle. The strongest difference detected from our analysis was ribosome biogenesis, where 41 genes were differentially expressed in both data sets. Under low light conditions, almost all the ribosome biogenesis genes were negatively differentially expressed, and all but two were upregulated at the end (12 and 21 h) of the infection cycle. This down regulation in low light may be due to decreased growth rates, as decreasing growth rate decreases ribosome biogenesis in *Saccharomyces cerevisiae* [44]. In contrast, protein synthesis is essential for new viral progeny, so much so that during Herpes Simplex Virus 1 infections, ribosome production still occurs late in the infection cycle while much of the other cell’s machinery has broken down [45]. This relationship between ribosome biogenesis and translation limits in low light infections has been hypothesized in cyanomyoviruses infecting the cyanobacterium *Synechococcus*. Shifting to higher light levels reduced the length of infections, with the host upregulating genes for ribosome biogenesis and translation [46]. If there is not enough ribosomal machinery within the infected cell to produce all of the required viral proteins for its infection cycle, the length of the cycle may increase. Alternatively, as the mechanism which triggers cell lysis is unknown in this system, the reduction in burst size could also be explained as not enough complete viruses were synthesized before cell lysis occurred.

### Potential ecological impact

**Fig 7:**
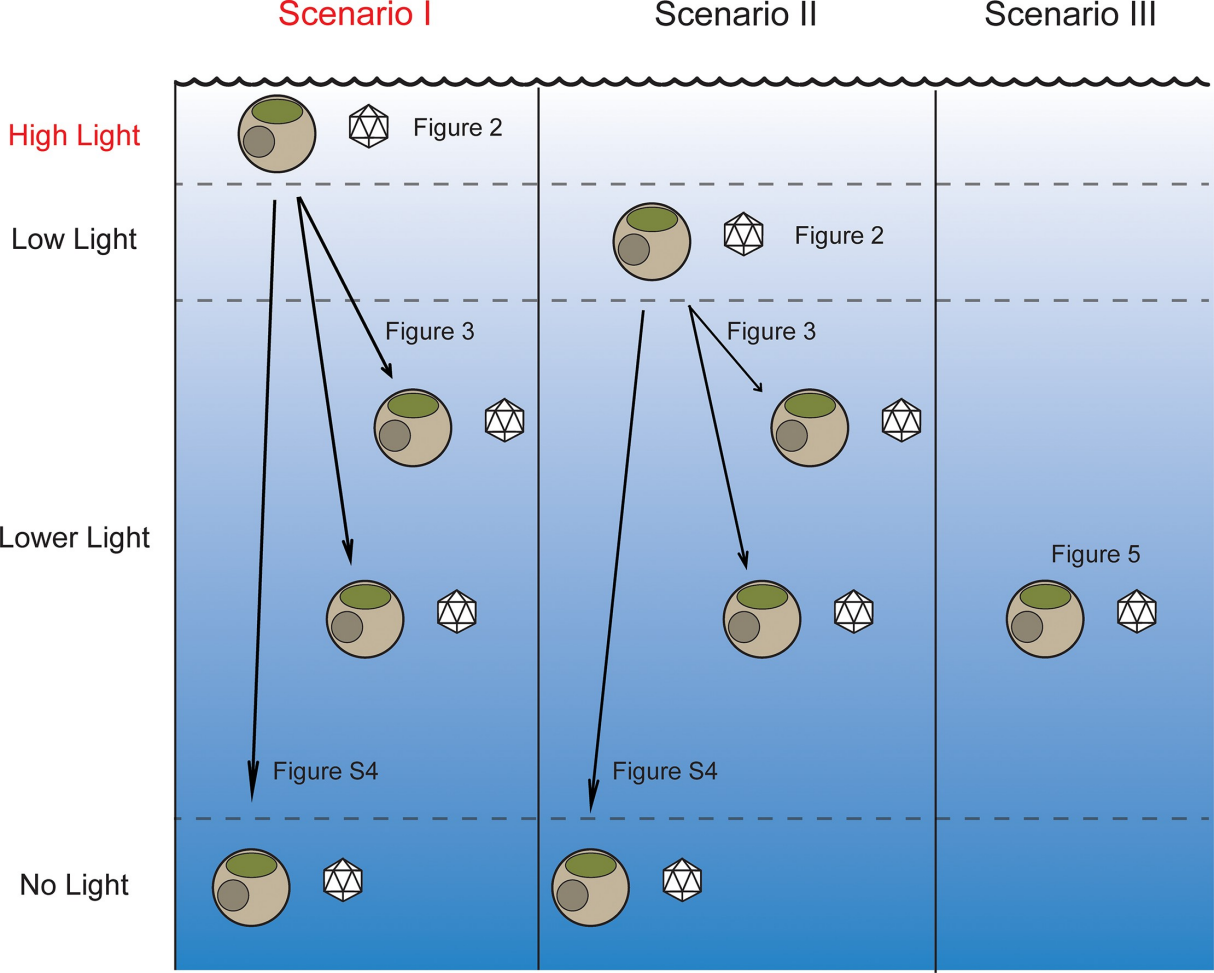
Ecological predictions of *Aureococcus* AaV interactions over the course of a bloom and effects of sinking. High light corresponds to 90 μmol photons m^-2^ s^-1^, low light corresponds to 30 μmol photons m^-2^ s^-1^, lower light corresponds to 15 and 5 μmol photons m^-2^ s^-1^.

Overall, our data contribute to an enhanced understanding of differences in infection dynamics during the course of *A*. *anophagefferens* blooms as well as highlight potential differences in cells transitioning to lower light while infected, which could also provide a framework for sinking cells. The role of viruses in sinking cells and carbon export is now being studied more thoroughly. Analyses of environmental and sequencing data collected from the TARA expeditions predicted that viruses may be important drivers of carbon export, potentially more so than organisms classically considered to be [47]. This is in agreement with previous data that viral infection increases rates of sinking in natural *Emiliania huxleyi* bloom mesocosoms [15], as well as in *Heterosigma akashiwo* culture experiments [48]. In the lab, *Aureococcus* settles during growth and phytoplankton populations during *A*. *anophagefferens* blooms have been shown to settle to the bottom of mesocosms [49], suggesting understanding the shift from one environment to another in this system is environmentally relevant. We presented three scenarios corresponding to changing environments encountered over the course of an entire bloom event (Fig 7). Surface viral infections are most productive during pre-bloom conditions (scenario I) where no reduction in light has occurred. While these cells sink, infections can occur while cells still have light, but not once complete darkness is achieved. As the cell concentration increases, the irradiance begins to decrease due to shading causing surface infections to take longer and reductions in viruses produced per cell to occur (scenario II). At the peak of the bloom (scenario III), light levels are low [27], and can severely limit growth. Virus infection dynamics are severely attenuated, but as the cellular concentrations begin to decrease, due to viral infection or other factors, virus infections begin to be more successful due to more light availability (scenario I & II), and with the higher cell densities early in the bloom, more contacts between viruses and the hosts can occur, potentially leading to bloom collapse [9].

Although irradiance levels vary considerably over the course of the bloom [27], many other variables that change throughout the bloom (including temperature, nitrogen and phosphorus [22]) which have the potential to influence infection success, should be considered. Reducing nutrients, such as nitrogen or phosphorus, have been shown to reduce the burst size and increase the time to lysis in other viruses [21, 50]. An additive effect was shown in the *Phaeocystis globosa* system, where suboptimal light and low phosphorus reduced the PgV burst size to less than ten infectious particles per cell [40]. As late in the bloom, *A*. *anophagefferens* appears to be phosphorus limited [51, 52], and the amount of light *A*. *anophagefferens* has is greatly reduced [27], could drastically alter the success of an *A*. *anophagefferens* infecting virus late in the bloom if additive like the *P*. *globosa* system. As these studies are conducted in the lab, more work is needed to estimate the production rate of viruses over the course of the bloom to determine how the dynamic abiotic factors influence the production of viruses, but these studies do provide evidence for the importance in considering abiotic factors in this bloom system. Also, future work understanding how the interplay between abiotic factors (*i*.*e*. low DIN:DON ratios), predators (such as viruses), and competing phytoplankton controls *A*. *anophagefferens* densities in areas where no blooms form will be important as *Aureococcus* spreads globally.

In summary, we showed the AaV infection cycle requires light to proceed, and is influenced by the past and current irradiance the host is in. The length of the infection cycle is increased when light is reduced, and the number of new viruses produced per cell is decreased in lower light, which we hypothesize is due in part to a reduced availability of translational machinery within the cell. We used the data generated in this study to provide an environmentally relevant framework that describes the potential importance of changing light levels on infections on a population level (shading due to high cell densities) and on an individual level (a sinking cell).

## Supporting information

S1 Fig Standard curve validating qPCR method to enumerate free AaV particles. Concentration of AaV particles was determined by epifluorescence microscopy, and concentration of plasmids was determined by conversion of DNA concentration to copy number. Points are for n = 3 biological replicates ± SD, with technical qPCR reaction replicates.
(TIF)

S2 Fig Comparison between total virus particle counts as determined by SYBR green epifluorescence microscopy and infectious particles determined by plaque assay or most probable number. For each, triplicate replicate cultures were infected, and once the cultures cleared, aliquots were taken for either type of enumeration. Plaque assays for each culture were done at two dilutions in duplicate. MPN assays had duplicate plates with 7 replicates per plate. There was a significant difference between the microscopy counts and the infectious particle counts (SYBR Green v. Plaque assay, pair t-test, p < 0.01; SYBR Green v. MPN, paired t-test, p < 0.001).
(TIF)

S3 Fig Growth curve of *A*. *anophagefferens* acclimated to three different irradiance levels. To the three irradiance levels: black line—low (30 μmol photons m^-2^ s^-1^), blue line–medium (60 μmol photons m^-2^ s^-1^), red line–high (90 μmol photons m^-2^ s^-1^Points are for n = 5 five biological replicates ± SD.
(TIF)

S4 Fig Burst Size v. Irradiance Level during the infection from distinct experiments (Table 1 and Fig 4B). 90 μmol photons m^-2^ s^-1^ acclimated cultures from each experiment.
(TIFF)

S5 Fig *Aureococcus anophagefferens* virus infection cycle dynamics with acclimation to varying irradiance levels and then transferred to the dark. *A*. *anophagefferens* host concentrations either uninfected (A) or infected (B), and C) major capsid protein (MCP) copies mL^-1^ over the course of the 5-day experiment. Cultures were infected on day 0, and transferred to the dark. Red lines are high irradiance acclimated cultures (90 μmol photons m^-2^ s^-1^), blue lines are medium irradiance acclimated cultures (60 μmol photons m^-2^ s^-1^), and black lines are low irradiance acclimated cultures (30 μmol photons m^-2^ s^-1^).All symbols are for n = 5 biological replicates ± SD. Control cultures not placed in the dark are shown in Fig 2.
(TIF)

S1 Table Adjusted p-values comparing differences in AaV concentration on Day three (Fig 1) based on irradiance level and viral enumeration method as determined by two-way ANOVA with post-hoc multiple comparisons being adjusted with Tukey’s HSD. High light acclimated are cultured in 90 μmol photons m^-2^ s^-1^, Medium light acclimated cultures are cultured in 60 μmol photons m^-2^ s^-1^, low light acclimated cultures are cultured in 30 μmol photons m^-2^ s^-1^.
(PDF)

S2 Table Adjusted p-values comparing differences in burst sizes based on irradiance level cells were transferred to after infection as determined by one-way ANOVA with post-hoc multiple comparisons being adjusted with Tukey’s HSD (Fig 3). Low light corresponds to acclimation irradiance levels of 30 μmol photons m^-2^ s^-1^, and high light corresponds to 90 μmol photons m^-2^ s^-1^.
(PDF)

S3 Table Forward Scatter (FSC-H) values for high (90 μmol photons m^-2^ s^-1^) and low (30 μmol photons m^-2^ s^-1^) light acclimated cultures either maintained at acclimating light or transitioned 5 μmol photons m^-2^ s^-1^ for one day. Standard deviation of each value is recorded within the parenthesis.
(PDF)

S4 Table Summary of genes used in analysis for differences between low light conditions (from [25]) and the infection cycle [11]. (PDF)

S5 Table KEGG pathways of *A*. *anophagefferens* genes differentially expressed in both the CCMP1850 transcriptome [25] and the CCMP1984 infection cycle transcriptome [11]. Columns denote whether all of these genes differentially expressed over the course of the infection cycle at every time point are overexpressed (+), underexpressed (-), or a mixed (+/-).
(PDF)
