# Supplementary figures and images for: Influence of light on the infection of Aureococcus anophagefferens CCMP 1984 by a “giant virus”
Source: PLoS One. 2020 Jan 3;15(1):e0226758. doi: 10.1371/journal.pone.0226758 (PMC6941929; doi:10.1371/journal.pone.0226758)

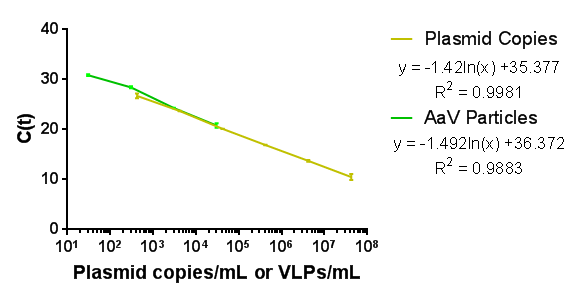

Supplement: S1 Fig — Concentration of AaV particles was determined by epifluorescence microscopy, and concentration of plasmids was determined by conversion of DNA concentration to copy number. Points are for n = 3 biological replicates ± SD, with technical qPCR reaction replicates. (TIF) [file pone.0226758.s001.tif]

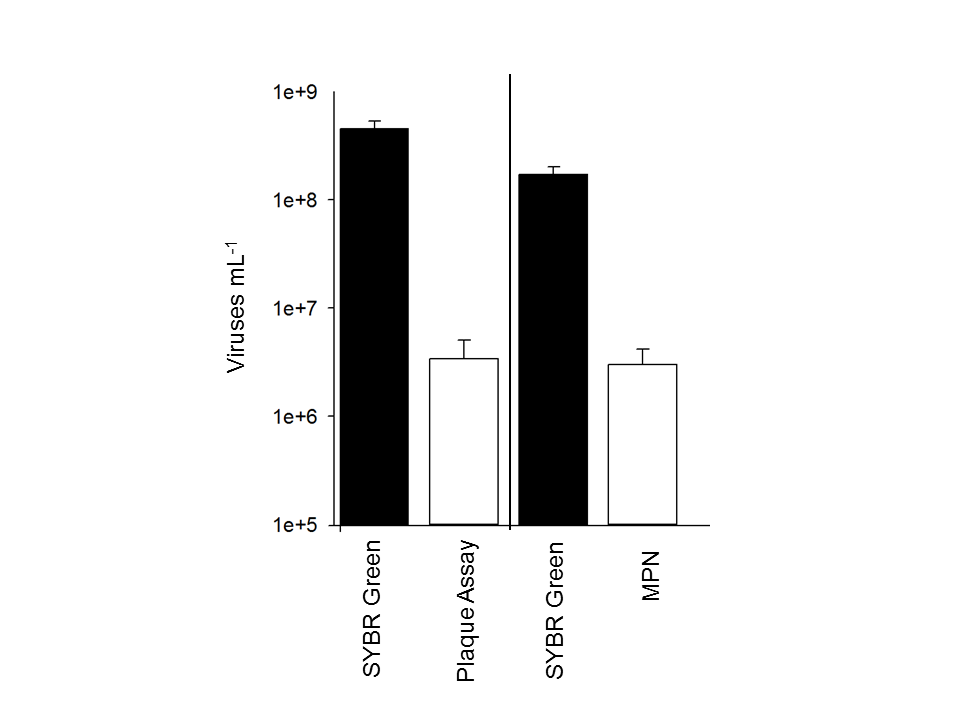

Supplement: S2 Fig — For each, triplicate replicate cultures were infected, and once the cultures cleared, aliquots were taken for either type of enumeration. Plaque assays for each culture were done at two dilutions in duplicate. MPN assays had duplicate plates with 7 replicates per plate. There was a significant difference between the microscopy counts and the infectious particle counts (SYBR Green v. Plaque assay, pair t-test, p < 0.01; SYBR Green v. MPN, paired t-test, p < 0.001). (TIF) [file pone.0226758.s002.TIF]

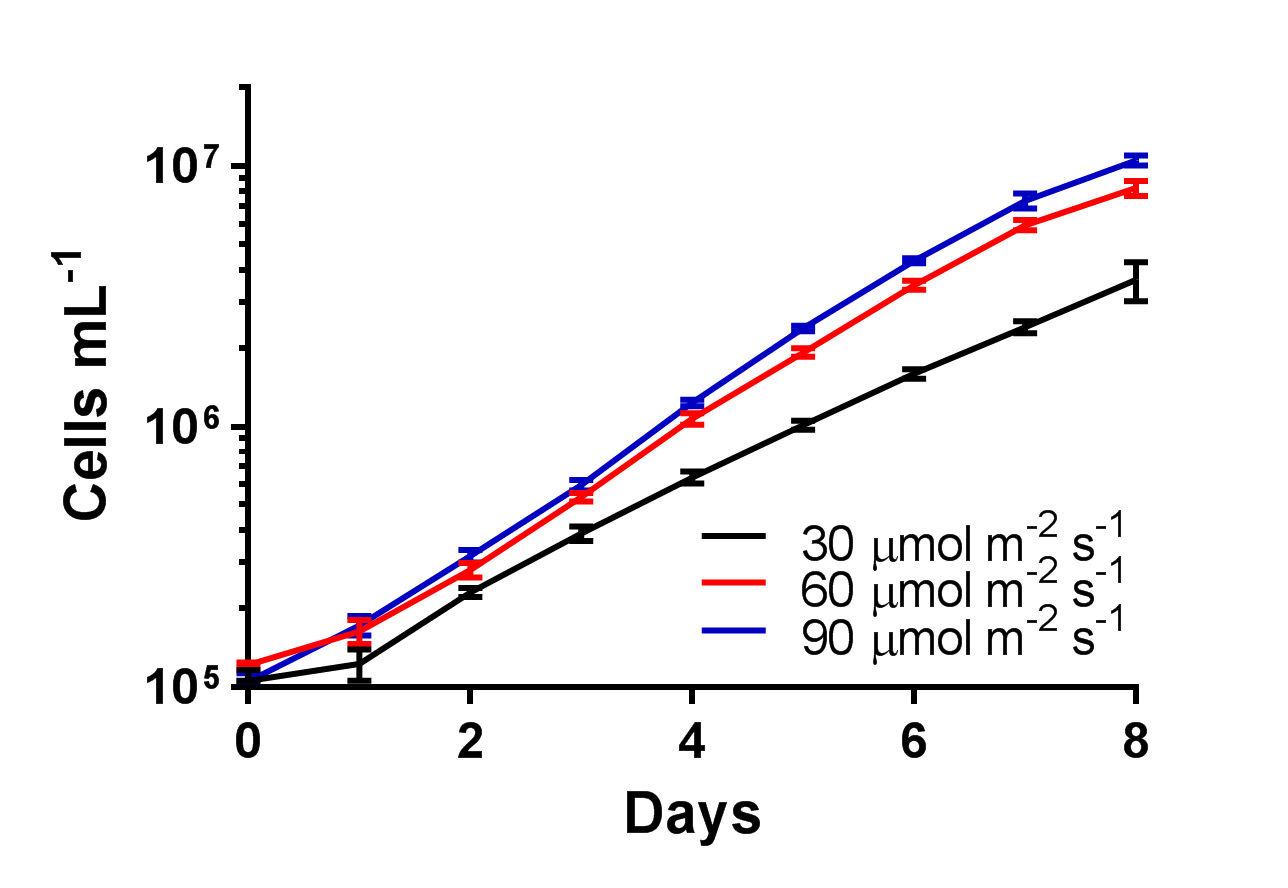

Supplement: S3 Fig — To the three irradiance levels: black line—low (30 μmol photons m-2 s-1), blue line–medium (60 μmol photons m-2 s-1), red line–high (90 μmol photons m-2 s-1Points are for n = 5 five biological replicates ± SD. (TIF) [file pone.0226758.s003.tif]

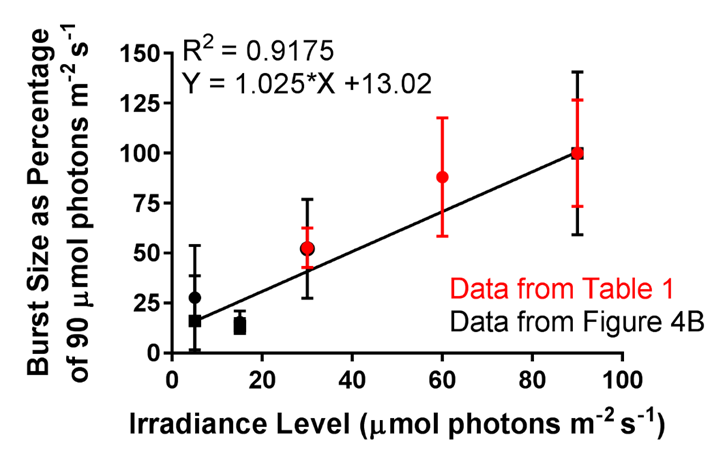

Supplement: S4 Fig — 90 μmol photons m-2 s-1 acclimated cultures from each experiment. (TIFF) [file pone.0226758.s004.tiff]

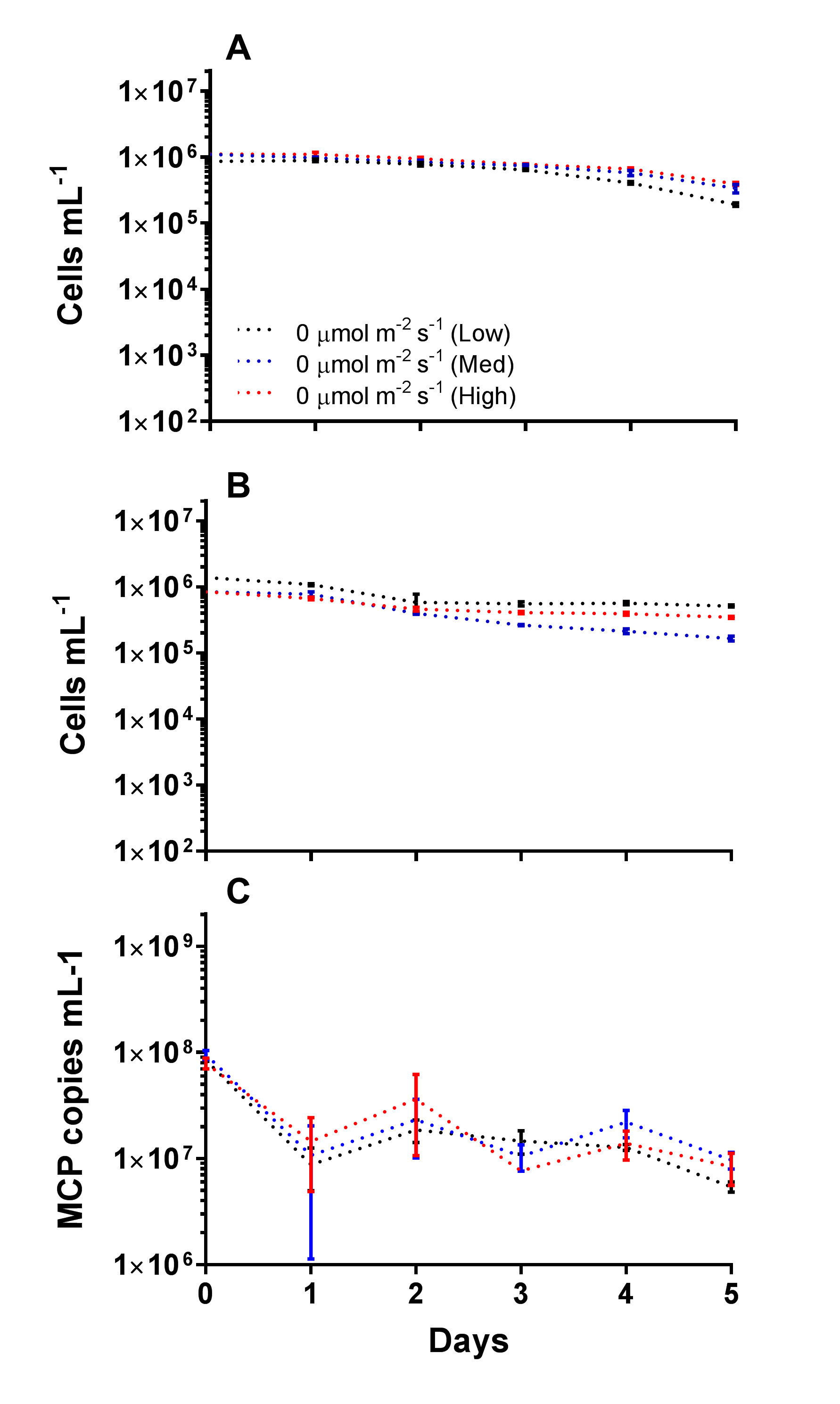

Supplement: S5 Fig — A. anophagefferens host concentrations either uninfected (A) or infected (B), and C) major capsid protein (MCP) copies mL-1 over the course of the 5-day experiment. Cultures were infected on day 0, and transferred to the dark. Red lines are high irradiance acclimated cultures (90 μmol photons m-2 s-1), blue lines are medium irradiance acclimated cultures (60 μmol photons m-2 s-1), and black lines are low irradiance acclimated cultures (30 μmol photons m-2 s-1).All symbols are for n = 5 biological replicates ± SD. Control cultures not placed in the dark are shown in Fig 2. (TIF) [file pone.0226758.s005.tif]
