# Supplementary material for: Influence of light on the infection of Aureococcus anophagefferens CCMP 1984 by a “giant virus”
Source: PLoS One. 2020 Jan 3;15(1):e0226758. doi: 10.1371/journal.pone.0226758 (PMC6941929; doi:10.1371/journal.pone.0226758)
Supplement: S2 Table — Low light corresponds to acclimation irradiance levels of 30 μmol photons m-2 s-1, and high light corresponds to 90 μmol photons m-2 s-1. (PDF) [file pone.0226758.s007.pdf]

S2 Table. Adjusted p-values comparing differences in burst sizes based on irradiance level cells were transferred to after infection as determined by one-way ANOVA with post-hoc multiple comparisons being adjusted with Tukey's HSD (Fig 3). Low light corresponds to acclimation irradiance levels of 30 µmol photons m^{-2} s^{-1}, and high light corresponds to 90 µmol photons m^{-2} s^{-1}.

		Low			High		
Acclimated Light	Shifted Light	30 µmol photons m^{-2} s^{-1}	15 µmol photons m^{-2} s^{-1}	5 µmol photons m^{-2} s^{-1}	90 µmol photons m^{-2} s^{-1}	15 µmol photons m^{-2} s^{-1}	5 µmol photons m^{-2} s^{-1}
Low	30 µmol photons m^{-2} s^{-1}	-	0.205	0.617	0.052	0.167	0.394
	15 µmol photons m^{-2} s^{-1}		-	0.966	<0.001	>0.999	>0.999
	5 µmol photons m^{-2} s^{-1}			-	0.001	0.939	0.997
High	90 µmol photons m^{-2} s^{-1}				-	<0.001	<0.001
	15 µmol photons m^{-2} s^{-1}					-	0.999
	5 µmol photons m^{-2} s^{-1}						-
