# Supplementary material for: Influence of light on the infection of Aureococcus anophagefferens CCMP 1984 by a “giant virus”
Source: PLoS One. 2020 Jan 3;15(1):e0226758. doi: 10.1371/journal.pone.0226758 (PMC6941929; doi:10.1371/journal.pone.0226758)
Supplement: S3 Table — Standard deviation of each value is recorded within the parenthesis. (PDF) [file pone.0226758.s008.pdf]

S3 Table. Forward Scatter (FSC-H) values for high (90 μmol photons m^{-2} s^{-1}) and low (30 μmol photons m^{-2} s^{-1}) light acclimated cultures either maintained at acclimating light or transitioned 5 μmol photons m^{-2} s^{-1} for one day. Standard deviation of each value is recorded within the parenthesis.

Pre-acclimation Light Level	90 μmol photons m^{-2} s^{-1}	90 μmol photons m^{-2} s^{-1}	30 μmol photons m^{-2} s^{-1}	30 μmol photons m^{-2} s^{-1}
Acclimation Light Level	90 μmol photons m^{-2} s^{-1}	5 μmol photons m^{-2} s^{-1}	30 μmol photons m^{-2} s^{-1}	5 μmol photons m^{-2} s^{-1}
Pre-acclimated FSC-H (Relative Units)	15.92 (0.05)	15.92 (0.05)	13.28 (0.06)	13.28 (0.06)
Acclimated FSC-H (Relative Units)	16.62 (0.82)	9.36 (0.22)	12.78 (0.06)	9.52 (0.19)
