# Supplementary material for: Influence of light on the infection of Aureococcus anophagefferens CCMP 1984 by a “giant virus”
Source: PLoS One. 2020 Jan 3;15(1):e0226758. doi: 10.1371/journal.pone.0226758 (PMC6941929; doi:10.1371/journal.pone.0226758)
Supplement: S4 Table — (PDF) [file pone.0226758.s009.pdf]

S4 Table. **Summary of genes used in analysis for differences between low light conditions (from [25]) and the infection cycle [11].**

		Number of genes with at least 1 significant significantly expressed (>1.5 fold) in Infection cycle transcriptome (Moniruzzaman et al. 2018)				
	# Genes differentially expressed in LowL (Frischkorn et al. 2014)	Upregulated / non-significant in all time points	Mixed / non – significant all time points	Downregulated / non-significant in all time points	# Genes with Kegg IDs	# Genes with Kegg Pathways
LowL upregulated	256	27	16	113	55	37
LowL downregulated	1268	221	22	351	159	39
