# Supplementary material for: Influence of light on the infection of Aureococcus anophagefferens CCMP 1984 by a “giant virus”
Source: PLoS One. 2020 Jan 3;15(1):e0226758. doi: 10.1371/journal.pone.0226758 (PMC6941929; doi:10.1371/journal.pone.0226758)
Supplement: S5 Table — Columns denote whether all of these genes differentially expressed over the course of the infection cycle at every time point are overexpressed (+), underexpressed (-), or a mixed (+/-). (PDF) [file pone.0226758.s010.pdf]

S5 Table. KEGG pathways of *A. anophagefferens* genes differentially expressed in both the CCMP1850 transcriptome [25] and the CCMP1984 infection cycle transcriptome [11].

Columns denote whether all of these genes differentially expressed over the course of the infection cycle at every time point are overexpressed (+), underexpressed (-), or a mixed (+/-).

	Down in LowL			Up in LowL				Down in LowL			Up in LowL		
KEGG Pathway	+	+/-	-	+	+/-	+	KEGG Pathway	+	+/-	-	+	+/-	+
ABC transporters			2		1	1	Glyoxylate and dicarboylate metabolism			1			
Amino sugar and nucleotide sugar metabolism	1		1		1	3	Metabolic pathways	7		7	4	2	14
Aminoacyl-tRNA biosynthesis	1					1	Monobactam biosynthesis			1			
Arachidonic acid metabolism				1			Nitrogen metabolism	1		1			
Arginine and Proline metabolism				1		1	One carbon pool by folate	1					
Ascorbate and aldarate metabolism						1	Pentose and glucuronate interconversions			1			1
Biosynthesis of amino acids	2			1		1	Pentose phosphate pathway				1		3
Biosynthesis of antibiotics	1		3	2		7	Peroxisome/Oxidative stress						2
Biosynthesis of secondary metabolites	3		2	2		4	Phagosome			2			1
Biotin metabolism						2	Phosphatidylinositol signaling system				1		
Carbon metabolism	1		1	2		3	Porphyrin and chlorophyll metabolism	1					
Cyanoamino acid metabolism			1				Protein Export					1	
Cysteine and methioine metabolism	1						Protein processing in endoplasmic recticulum	3					
DNA replication			3			1	Purine metabolism			3	1		
Endocytosis	1		3			1	Ribosome				1		
Ether lipid metabolism	1						Ribosome biogenesis in eukaryotes	13		2			
Fatty acid biosynthesis	2					3	RNA degradation			2		1	2
Fatty acid metabolism	2					3	RNA polymerase	1		1			
Fructose and mannose metabolism	1			1			RNA transport	2					2
Galactose metabolism	1					1	Selenocompound metabolism	1		1			
Glutathione metabolism				2			Spliceosome	3					1
Glycerolipid metabolism			1			2	Starch and sucrose metabolism	1		1			
Glycerophospholipid metabolism			2				Sulfur metabolism	1		1			
Glycine, serine and threonine metabolism				1			Terpenoid backbone biosynthesis						1
Glycolysis/Gluconeogenesis	1					2	Ubiquitin mediated proteolysis			2			
Glycosaminoglycan degradation	1						Valine, Leucine, and isoleucine biosynthesis				1		
