# Supplementary material for: Influence of light on the infection of Aureococcus anophagefferens CCMP 1984 by a “giant virus”
Source: PLoS One. 2020 Jan 3;15(1):e0226758. doi: 10.1371/journal.pone.0226758 (PMC6941929; doi:10.1371/journal.pone.0226758)
Supplement: S1 Table — High light acclimated are cultured in 90 μmol photons m-2 s-1, Medium light acclimated cultures are cultured in 60 μmol photons m-2 s-1, low light acclimated cultures are cultured in 30 μmol photons m-2 s-1. (PDF) [file pone.0226758.s006.pdf]

S1 Table. Adjusted p-values comparing differences in AaV concentration on Day three (Fig 1) based on irradiance level and viral enumeration method as determined by two-way ANOVA with post-hoc multiple comparisons being adjusted with Tukey's HSD.

High light acclimated are cultured in 90 μmol photons m^{-2} s^{-1}, Medium light acclimated cultures are cultured in 60 μmol photons m^{-2} s^{-1}, low light acclimated cultures are cultured in 30 μmol photons m^{-2} s^{-1}.

Enumeration Method		qPCR			SYBR			MPN		
	Acclimation	High	Med	Low	High	Med	Low	High	Med	Low
qPCR	High	-	0.371	0.939	<0.001	<0.001	<0.001	<0.001	<0.001	<0.001
	Med		-	0.966	<0.001	<0.001	<0.001	<0.001	<0.001	<0.001
	Low			-	<0.001	<0.001	<0.001	<0.001	<0.001	<0.001
SYBR	High				-	0.999	0.996	0.14	0.088	0.093
	Med					-	>0.999	0.026	0.012	0.013
	Low						-	0.02	0.001	0.01
MPN	High							-	>0.999	>0.999
	Med								-	>0.999
	Low									-
